# General Maintenance and Reactivation of iSLK Cell Lines

**DOI:** 10.21769/BioProtoc.5334

**Published:** 2025-06-05

**Authors:** Ariana C. Calderón-Zavala, Aaron S. Mendez, Ekaterina E. Heldwein

**Affiliations:** 1 Department of Molecular Biology and Microbiology, Tufts University School of Medicine, Boston, MA, USA; 2 Graduate Program in Molecular Microbiology, Graduate School of Biomedical Sciences, Tufts University School of Medicine, Boston, MA, USA

**Keywords:** KSHV, HHV-8, iSLK-BAC16 cell lines, iSLK cell culture, KSHV reactivation, Doxycycline

## Abstract

Since the establishment of the iSLK-BAC16 cell culture system, iSLK-BAC16 cells and their derivatives have been widely used for Kaposi’s sarcoma-associated herpesvirus (KSHV) studies. However, iSLK-BAC16 cells can be difficult to work with, in part due to the lack of standardized protocols and conflicting troubleshooting suggestions. Here, we describe the protocol for general iSLK-BAC16 cell culture and reactivation, which induces lytic KSHV replication and virion production. This protocol achieves robust levels of KSHV reactivation in our hands and can be readily used for studies of KSHV lytic infection mechanisms.

Key features

• This protocol describes methods for culturing and antibiotically selecting iSLK-BAC16 cells for robust KSHV reactivation.

• Use of flow cytometry to quantify KSHV reactivation rates.

• Innovative use of automated plate readers to assess KSHV reactivation.

Graphical overview

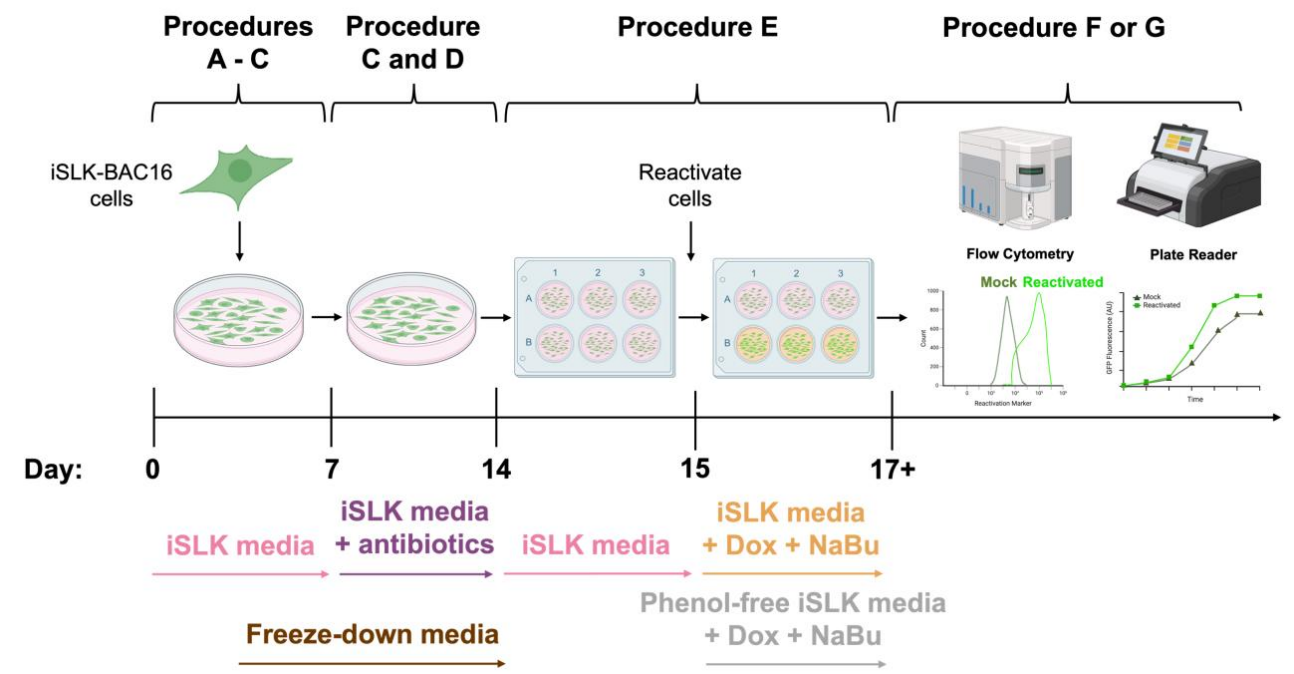

**Schematic overview of the procedures used for general iSLK-BAC16 cell culture and Kaposi’s sarcoma-associated herpesvirus (KSHV) reactivation of iSLK-BAC16 cells.** A typical timeline of iSLK-BAC16 cell culture, antibiotic selection, KSHV reactivation, flow cytometry quantification, and plate reader assessment of KSHV lytic replication. Corresponding media requirements are denoted under the respective procedures. iSLK-BAC16 = doxycycline-inducible endothelial cells harboring the KSHV genome on an artificial bacterial chromosome; Dox = doxycycline; NaBu = sodium butyrate.

## Background

Kaposi’s sarcoma-associated herpesvirus (KSHV), also known as human herpesvirus-8 (HHV-8), is a member of the Gammaherpesvirinae subfamily of the Orthoherpesviridae family, commonly known as herpesviruses. KSHV can infect lymphocytes and endothelial cells, causing Kaposi’s sarcoma (KS), primary effusion lymphomas (PELs), and multi-centric Castleman’s disease [1–4]. Mechanisms of latent and lytic KSHV infection are commonly studied in in vitro cell culture using the doxycycline-inducible SLK (iSLK) cell culture system because its design allows for a tight control of KSHV latent and lytic replication stages. iSLK cells originate from uninfected SLK (endothelial) cells, derived from a patient with a gingival KS lesion [5]. These iSLK cells were latently infected with rKSHV.219 [6], a KSHV strain isolated from JSC-1, a PEL-derived KSHV-positive cell line [7], and selected for successful integration of the rKSHV.219 genome using puromycin [8]. To tightly control the switch from KSHV latency into the lytic program, iSLK.219 cells were engineered to express RTA (the KSHV lytic transactivator) under the control of a tetracycline (tet) operator (selected with hygromycin). RTA expression can be induced using the reverse, tetracycline-controlled transactivator (rtTA) Tet-On system (selected with G418). Upon doxycycline (Dox) treatment, RTA gene expression is initiated, and the KSHV lytic gene expression program is activated, resulting in robust viral gene expression and viral replication [8]. Of the established iSLK cell lines, the most commonly used are iSLK-BAC16 cells and their derivatives, which harbor the rKSHV.219 genome on a bacterial artificial chromosome (BAC16) but retain the Dox-inducible RTA system [9]. iSLK-BAC16-cells constitutively express GFP as a marker for the Dox-inducible RTA system, yet can be engineered to express additional fluorescent reporters [10–12]. The use of fluorescent reporters in iSLK-BAC16 cells facilitates monitoring cells during antibiotic selection to enrich for those containing the KSHV genome.

The present protocol describes the procedures for general maintenance of iSLK-BAC16 cell cultures and appropriate antibiotic selection of iSLK-BAC16 cells harboring the Dox-inducible RTA system to achieve robust KSHV reactivation (~90%), as opposed to the large variation (40%–80%) observed in previous studies [9–10,13]. Although this protocol was developed on the basis of methods described in [9–11], it provides details lacking in prior protocols, including cell density requirements prior to and during antibiotic selection, target antibiotic concentrations necessary to maintain the Dox-inducible RTA system, and optimal cell density requirements for robust KSHV reactivation. Using an automated plate reader and flow cytometry, we assessed and quantified KSHV reactivation rates by detecting fluorescent reporters for latent and lytic infection [9–11]. The procedures in this protocol can be applied to other iSLK-BAC16 cell derivatives.

## Materials and reagents

**Biological materials**

1. iSLK-BAC16 (a gift from Jae Jung, Cleveland Clinic, Lerner Research Institute)

a. Encodes a constitutively expressed GFP transgene (green)

2. iSLK-BAC16-mIFP2-ORF57pr-EGFP cells [10] (a gift from Britt Glaunsinger, University of California, Berkeley)

a. Encodes a constitutively expressed mIFP2 transgene (far-red)

b. Encodes an EGFP transgene (green) driven by the immediate early viral gene promoter, ORF57, and expressed upon Dox induction

3. iSLK-BAC16-HaloTag-ORF68 cells [11] (a gift from Britt Glaunsinger, University of California, Berkeley)

a. Encodes a constitutively expressed GFP transgene (green)

b. Encodes an N-terminal HaloTag fused to the early viral gene, ORF68

c. Upon Dox induction, ORF68-Halo is expressed, which is detected by the addition of Janelia Fluor-646 HaloTag ligand (orange or far-red)

**Reagents**

1. Fetal bovine serum (FBS), heat-inactivated (Biowest, catalog number: S1680), store at -20 °C

2. GlutaMAX, 100× (Gibco, catalog number: 35050061), store at 4 °C

3. Penicillin-streptomycin, 100× (Cytiva, catalog number: SV30010), store at 4 °C

4. DMEM, high glucose, (+) L-glutamine, (-) sodium pyruvate (Gibco, catalog number: 11965-118), store at 4 °C

5. Trypsin, 0.05%, 1× (Cytiva, catalog number: SH30236.01), store at -20 °C

6. Phosphate buffered saline (PBS) 1× (Cytiva, catalog number: SH30256.01), store at room temperature

7. Trypan blue solution (Cytiva, CAS number: 72-57-1), store at room temperature

8. Dimethyl sulfoxide (DMSO) (Corning, catalog number: 25-950-CQC), store at room temperature

9. Isopropanol (Fisher Scientific, CAS number: 67-63-0), store at room temperature

10. Hygromycin B in PBS (AG Scientific, CAS number: 31282-04-9), store at 4 °C

11. G418 (VWR, CAS number: 108321-42-2), store at 4 °C

12. Puromycin dihydrochloride from *Streptomyces alboniger* (Sigma-Aldrich, CAS number: 58-58-2), store at 4 °C

13. Doxycycline hydrochloride (Fisher Scientific, CAS number: 10592-13-9), store at 4 °C

14. Doxycycline hyclate (Tocris Bioscience, CAS number: 24390-14-5), store at 4 °C

15. Sodium butyrate (Fischer Scientific, CAS number: 156-54-7), store at 4 °C

16. Janelia Fluor 646 Halo Ligand, 200 μM (Promega, catalog number: GA1120), store in amber tubes at -20 °C

17. Paraformaldehyde (PFA) in PBS, 4% (Santa Cruz Biotechnology, CAS number: 30525-89-4), store at 4 °C

18. Phenol-free DMEM, high glucose, HEPES, (+) L-glutamine, (-) sodium pyruvate (Gibco, catalog number: 21-063-029), store at 4 °C

19. Bleach (Clorox, catalog number: 30966), store at room temperature

**Solutions**

1. iSLK general cell culture media (iSLK media) (see Recipes)

2. G418 stock solution in PBS, 10 mg/mL (see Recipes)

3. Puromycin dihydrochloride stock solution in PBS, 1 mg/mL (see Recipes)

4. iSLK selection media (iSLK media + antibiotics) (see Recipes)

5. Freeze-down media (see Recipes)

6. Doxycycline hydrochloride or hyclate stock solution in PBS, 5 mg/mL (see Recipes)

7. Sodium butyrate stock solution in PBS, 1 M (see Recipes)

8. Janelia Fluor 646 Halo Ligand, 20 μM (see Recipes)

9. Phenol-free iSLK cell culture media (phenol-free iSLK media) (see Recipes)

10. Bleach, 20% (see Recipes)

**Recipes**

**1. iSLK general cell culture media (iSLK media)**

Store at 4 °C for up to 3 months.

**Table:** 

Reagent	Final concentration	Volume
DMEM, high glucose, (+) L-glutamine, (-) sodium pyruvate	1×	500 mL
FBS, heat-inactivated	10%	50 mL
GlutaMAX, 100×	1×	5 mL
Penicillin-streptomycin, 100×	1×	5 mL

**2. G418 stock solution in PBS (10 mg/mL)**

Store at -20 °C for up to 12 months.

**Table:** 

Reagent	Final concentration	Quantity or Volume
G418	10 mg/mL	10 mg
PBS	1×	1 mL

Filter-sterilize using a 0.22 μm syringe filter. Store at -20 °C in 1 mL aliquots. Avoid repeated freeze-thaw cycles.

**3. Puromycin dihydrochloride stock solution in PBS (1 mg/mL)**

Store at -20 °C for up to 12 months.

**Table:** 

Reagent	Final concentration	Quantity or Volume
Puromycin dihydrochloride	1 mg/mL	1 mg
PBS	1×	1 mL

Filter-sterilize using a 0.22 μm syringe filter. Store at -20 °C in 500 μL aliquots. Avoid repeated freeze-thaw cycles.

**4. iSLK selection media (iSLK media + antibiotics)**

Store at 4 °C for up to 3 months.

**Table:** 

Reagent	Final concentration	Volume
DMEM, high glucose, (+) L-glutamine, (-) sodium pyruvate	1×	500 mL
FBS, heat-inactivated	10%	50 mL
GlutaMAX, 100×	1×	5 mL
Penicillin-streptomycin, 100×	1×	5 mL
Hygromycin B in PBS, 50 mg/mL	1 mg/mL	10 mL
G418, 10 mg/mL	50 μg/mL	2.5 mL
Puromycin dihydrochloride, 1 mg/mL	1 μg/mL	500 μL

**5. Freeze-down media**

Store at 4 °C for up to 3 months.

**Table:** 

Reagent	Final concentration	Volume
FBS, heat-inactivated	1×	4.5 mL
DMSO	10%	500 μL

**6. Doxycycline hydrochloride or hyclate stock solution in PBS, 5 mg/mL**

Store at -20 °C for up to 12 months.

**Table:** 

Reagent	Final concentration	Quantity or Volume
Doxycycline hydrochloride or hyclate	5 mg/mL	5 mg
PBS	1×	1 mL

Filter-sterilize using a 0.22 μm syringe filter. Store at -20 °C in 50 μL aliquots.

**7. Sodium butyrate stock solution in PBS, 1 M**

Store at -20 °C for up to 12 months.

**Table:** 

Reagent	Final concentration	Quantity or Volume
Sodium butyrate	1 M	110.09 mg
PBS	1×	1 mL

MW: 110.09 g/mol. Filter-sterilize using a 0.22 μm syringe filter. Store at -20 °C in 50 μL aliquots.

**8. Janelia Fluor 646 Halo Ligand, 20 μM**

**Table:** 

Reagent	Final concentration	Quantity or Volume
Janelia Fluor 646 Halo Ligand, 200 μM	20 μM	10 μL
DMSO	1×	90 μL

Halo Ligand is sensitive to light and freeze-thawing. Store in amber tubes at -20 °C in 10 μL aliquots for up to 6 months.

**9. Phenol-free iSLK cell culture media (phenol-free iSLK media)**

Store at 4 °C for up to 3 months.

**Table:** 

Reagent	Final concentration	Volume
Phenol-free DMEM, high glucose, HEPES, (+) L-glutamine, (-) sodium pyruvate	1×	500 mL
FBS, heat-inactivated	10%	50 mL
GlutaMAX, 100×	1×	5 mL
Penicillin-streptomycin, 100×	1×	5 mL

**10. Bleach, 20%**

Prepare fresh before use. Store at room temperature for up to 24 hours.

**Table:** 

Reagent	Final concentration	Volume
Bleach	20%	10 mL
Deionized water	1×	40 mL

**Laboratory supplies**

1. Aspirating pipettes, 2 mL (Celltreat, catalog number: 229262)

2. Pipettes, 5 mL (Celltreat, catalog number: 667205B)

3. Pipettes, 10 mL (Celltreat, catalog number: 667210B)

4. Pipettes, 25 mL (Celltreat, catalog number: 667225B)

5. Filter pipette tips, 10 μL (USA Scientific, catalog number: 1120-3810)

6. Filter pipette tips, 200 μL (USA Scientific, catalog number: 1120-8810)

7. Filter pipette tips, 1,000 μL (USA Scientific, catalog number: 1122-1830)

8. Pipette, P1000 (ErgoOne, catalog number: 7110-1000)

9. Pipette, P200 (ErgoOne, catalog number: 7100-2200)

10. Pipette, P10 (ErgoOne, catalog number: 7100-0510)

11. Centrifuge tubes, 15 mL (Celltreat, catalog number: 229411)

12. Centrifuge tubes, 50 mL (Celltreat, catalog number: 229421)

13. 1 mL Luer-Lok syringe (BD, catalog number: 309628)

14. Syringe filter, 0.22 μm PVDF (Foxx Life Sciences, catalog number: 378-2415-OEM)

15. 150 mm × 20 mm (15 cm) plate, tissue culture treated (Celltreat, catalog number: 229620)

16. 100 mm × 20 mm (10 cm) plate, tissue culture treated (Celltreat, catalog number: 229651)

17. 6-well plate, tissue culture treated (Celltreat, catalog number: 229106)

18. 96-well plate, tissue culture treated, black (Greiner Bio-One, catalog number: 655090)

19. Cryogenic vials, 1 mL (Globe Scientific, catalog number: 3001)

20. Round-bottom tubes, 5 mL (Falcon, catalog number: 352052)

21. Mr. Frosty freezing container (Thermo Scientific, catalog number: 5100-0001)

22. Cell counting (hemocytometer) chamber slides (Invitrogen, catalog number: C10228)

23. Microcentrifuge tubes, 1.5 mL (BioPlas, catalog number: 4030)

## Equipment

1. CO_2_ cell culture incubator (Thermo Scientific, model: Heracell VIOS 160i)

2. Biosafety cabinet (Nuaire, model: NU-425-400)

3. 37 °C water bath (Fisher Scientific, model: Isotemp 228)

4. Benchtop centrifuge (Thermo Scientific, model: ST Plus Series)

5. -80 °C freezer (PHCbi, model: MDF-DU703VH-PA)

6. Liquid nitrogen storage tank (Thermo Scientific, model: Cryoplus 2)

7. Fluorescent microscope (Leica, model: DM IL LED)

8. Analytical scale (Mettler Toledo, model: X5R105DU)

9. Automated cell counter (Invitrogen, model: Countess II)

10. Flow cytometer (Thermo Scientific, model: Attune Cytpix)

11. Automated plate reader (Tecan, model: Spark)

## Software and datasets

1. Leica Application Suite X (LAS X) software (Leica, version 3.7.5.24914)

2. Attune cytometric software (Thermo Scientific, version 6.21)

3. FlowJo software (BD, version 10.10)

4. Prism software (GraphPad, version 10.4.1)

## Procedure

**A. Thawing iSLK cells**

*Notes:*

*1. All steps are performed in a laminar flow hood unless otherwise noted.*

*2. iSLK cells are frozen down at 2–5 × 10^6^ cells/mL in FBS + 10% DMSO (not iSLK media) to prevent ice formation. Once cells have thawed, the addition of iSLK media dilutes this solution. It is then replaced with fresh iSLK media for general cell culture.*

1. Prepare the tissue culture hood by cleaning surfaces with 70% ethanol.

2. Prepare iSLK media (see Recipe 1) and warm to 37 °C.

3. Retrieve cells (frozen down at 2–5 × 10^6^ cells/mL) from liquid nitrogen storage (-160 °C) and thaw to 37 °C.

*Note: Cells in a 37 °C water bath take roughly 5 min to thaw.*

4. Resuspend ~1 mL of cells from the cryo-vial in 9 mL of iSLK media in a 15 mL centrifuge tube.

**Critical:** Do not resuspend cells in iSLK selection media as cells must recover from being stored in liquid nitrogen (-160 °C).

5. Centrifuge cells at 500× *g* for 5 min.

6. Keeping the cell pellet intact, carefully aspirate the media using a 2-mL aspirating pipette.

7. Resuspend the cell pellet in 10 mL of iSLK media.

8. Label 10-cm plates: iSLK cell line (reporter system), passage number, thaw date, initials.

9. Plate resuspended cells in 10-cm plates and incubate at 37 °C with 5% CO_2_.

a. Swirl the cell suspension in the plates four times in a side-to-side motion and then four times in an up-and-down motion to evenly distribute cells.

b. The cell density plated should be 3.5–8.8 × 10^4^ cells/cm^2^.

**B. Passaging iSLK cells**

*Notes:*

*1. All steps are performed in a laminar flow hood unless otherwise noted.*

*2. iSLK cells are generally cultured and maintained with 10 mL of iSLK media in a 10-cm plate at 37 °C under 5% CO_2_. Once cells have been appropriately selected with antibiotics (see section D), passage and maintain cells in iSLK selection media. Once iSLK cells have reached ≥80% confluency (1.4–1.5 × 10^5^ cells/cm^2^), passage cells every 2–3 days (1:4 or 1:5 split), maintaining a minimum of 20% confluency (3.5–8.8 × 10^4^ cells/cm^2^). Keep cells in culture for up to one month (maximum of 12–14 passages), as described below. After one month, restart the culture from a frozen stock. The following protocol is for general iSLK cell culture using 10-cm plates. Adjust the volume of reagents in proportion to the bottom area of the plate, as shown by examples in Table 1.*

**Table 1.** General iSLK cell culture reagents by plate size

Plate type	6-well plate	10-cm plate	15-cm plate
Number of cells needed to seed 20% confluency	~3 × 10^5^ cells/mL	~2 × 10^6^ cells/mL	~5 × 10^6^ cells/mL
Volume of PBS needed to wash cells	1 mL	5 mL	10 mL
Volume of trypsin needed to detach cells	1 mL	1 mL	4 mL
Volume of iSLK media needed to inactivate trypsin	1 mL	9 mL	12 mL
Total volume of iSLK media	3 mL	10 mL	16 mL

1. Prepare the tissue culture hood by cleaning surfaces with 70% ethanol.

2. Prewarm iSLK media (see Recipe 1) or iSLK selection media (see Recipe 4) and trypsin to 37 °C.

3. Retrieve cells from the 37 °C incubator and check confluency under the microscope.

4. If cells are ≥80% confluent, aspirate the cell media using a 2-mL aspirating pipette.

**Critical:** Cells must be ≥80% confluent (1.4–1.5 × 10^5^ cells/cm^2^) during initial antibiotic selection to mitigate excessive cell death and ensure sufficient viable cells are present. This ensures the successful selection and survival of cells (see section D). If cells have not reached ≥80% confluency and 2–3 days have passed, replace the media.

5. Wash cells 1× with PBS (see Table 1).

6. Aspirate the PBS from plates using a 2-mL aspirating pipette.

7. Trypsinize cells for 5 min in the 37 °C incubator. Be sure to coat the entire plate with trypsin (see Table 1).

8. Once cells have detached from the plate, resuspend the cells in iSLK media (see Table 1). Ensure cells are fully resuspended and no cell clumps are visible.

9. Transfer the cell suspension into a 15- or 50-mL centrifuge tube.

10. In an Eppendorf tube, mix 10 μL of cell suspension with 10 μL of trypan blue. Add 10 μL of this mixture into a hemocytometer slide and count the number of live cells.

11. Based on the desired cell density, plate the corresponding volume of cell suspension into new, labeled plates. Swirl the cell suspension in the plates four times in a side-to-side motion and then four times in an up-and-down motion to evenly distribute cells.

12. Incubate cells at 37 °C with 5% CO_2_.

**Critical:** Do not seed cells below 20% confluency as this will lead to unusual growth phenotypes and potential cell death. However, do not allow cells to grow past 100% confluency because an evenly spread monolayer is important for proper cell maintenance and achieving robust KSHV reactivation in the future.

**C. Freezing down iSLK cells**

*Notes:*

*1. All steps are performed in a laminar flow hood unless otherwise noted.*

*2. Expand iSLK cells into 15-cm plates before and after antibiotic selection at 37 °C under 5% CO_2_. Once iSLK cells have reached ≥80% confluency (1.4–1.5 × 10^5^ cells/cm^2^) and are growing as expected, freeze down cells as described below. For every 15-cm plate of cells, prepare 5 mL of freeze-down media and five cryo-vials. Each cryo-vial should contain a cell density of 2–5 × 10^6^ cells/mL.*

1. Prepare the tissue culture hood by cleaning surfaces with 70% ethanol.

2. Prepare freeze-down media (see Recipe 5).

3. Label cryo-vials with ethanol-resistant marker: **iSLK cell line** (+ or – antibiotics), **passage number, initials**, and **date**.

4. Retrieve cells from the 37 °C incubator and check confluency under the microscope.

5. Aspirate the cell media using a 2-mL aspirating pipette.

6. Wash cells once with 10 mL of PBS.

7. Aspirate the PBS from the plates using a 2-mL aspirating pipette.

8. Trypsinize cells with 4 mL of trypsin for 5 min in the 37 °C incubator. Be sure to coat the entire plate.

9. Once cells have detached from the plate, resuspend cells in 12 mL of iSLK media. Ensure cells are fully resuspended and no cell clumps are visible.

10. Transfer the cell suspension into a 50 mL centrifuge tube.

11. Centrifuge the cells at 500× *g* for 5 min.

12. Keeping the cell pellet intact, carefully aspirate the media using a 2-mL aspirating pipette.

13. Resuspend the cell pellet in freeze-down media.

14. Aliquot 1 mL of cells (2–5 × 10^6^ cells/mL) into each labeled cryo-vial.

15. Place cryo-vials in a Mr. Frosty container filled with isopropanol.

16. Store cells in a Mr. Frosty in the -80 °C freezer for 1–2 days, then transfer cryo-vials to -160 °C liquid nitrogen storage for long-term storage.

**D. Antibiotically selecting iSLK cells**

*Notes:*

*1. All steps are performed in a laminar flow hood unless otherwise noted.*

*2. Proper and constant antibiotic selection of iSLK cells is critical for maintaining the Dox-inducible RTA system and achieving robust levels of KSHV reactivation as antibiotic withdrawal leads to loss of the Dox-inducible RTA system and KSHV latency in ~1–2 weeks. Once iSLK cells have reached ≥80% confluency (1.4–1.5 × 10^5^ cells/cm^2^, typically 1–2 days after thawing), begin antibiotic selection. Maintaining high levels of cell density during initial antibiotic selection mitigates notable cell death (cell viability <50%) and ensures sufficient numbers of viable cells, leading to successful selection and survival of cells harboring the KSHV genome. During antibiotic selection, if cells are ≥80% confluent (1.4–1.5 × 10^5^ cells/cm^2^) but notable cell death is observed (cell viability <50%), split cells 1:2 using iSLK selection media. Once little to no cell death is observed (cell viability ≥75%), split cells as normal (1:4 or 1:5) using iSLK selection media. Once notable cell death is no longer observed, and cells are growing under antibiotic selection, cells are ready to reactivate. iSLK cells must be grown under constant antibiotic selection until seeding for reactivation. The following protocol is for general iSLK cell culture using 10-cm plates. Adjust the volume of reagents in proportion to the bottom area of the plate, as shown by examples in Table 1.*

1. Prepare the tissue culture hood by cleaning surfaces with 70% ethanol.

2. Prepare antibiotic stock solutions (see Recipes 2 and 3).

3. Prepare iSLK selection media (see Recipe 4) and warm to 37 °C.

4. Retrieve cells from the 37 °C incubator and check confluency under the microscope.

**Critical:** Cells must be ≥80% confluent (1.4–1.5 × 10^5^ cells/cm^2^) during initial antibiotic selection to mitigate excessive cell death (cell viability <50%) and ensure sufficient viable cells are present. This ensures the successful selection and survival of cells.

5. Wash cells once with PBS (see Table 1).

6. Add iSLK selection media to the cells and incubate at 37 °C with 5% CO_2_ (see Table 1).

7. Check cells every day during antibiotic selection and passage as necessary using iSLK selection media (see section B).

**Critical:** If cells are ≥80% confluent (1.4–1.5 × 10^5^ cells/cm^2^) but notable cell death (cell viability <50%) is observed, split 1:2 using iSLK selection media. Once little to no cell death is observed (cell viability ≥75%), split cells 1:4 or 1:5 using iSLK selection media every 2–3 days. Cell viability can be assessed under the microscope (dying iSLK cells round up and detach from the plate) and quantified on a hemocytometer using trypan blue staining.

a. If severe cell death is observed (cell viability <30%), antibiotic concentrations may need to be adjusted (see Troubleshooting).

8. Expand cells into 15-cm plates to freeze down antibiotically selected iSLK cells (see section C).

9. Maintain cells under constant antibiotic selection until seeding for reactivation.

**Critical:** Antibiotic withdrawal leads to loss of the Dox-inducible RTA system and KSHV latency in ~1–2 weeks.

**E. KSHV reactivation of iSLK cells**

*Notes:*

*1. All steps are performed in a laminar flow hood unless otherwise noted.*

*2. Proper antibiotic selection of iSLK cells ensures they contain the Dox-inducible RTA system, which harbors the KSHV genome. Once iSLK cells have successfully undergone antibiotic selection (see section D), they can be seeded for KSHV reactivation. For robust KSHV reactivation, doxycycline hydrochloride (or doxycycline hyclate; see Figure S1) and sodium butyrate (NaBu) must be used together. The following protocol is for KSHV reactivation of iSLK cells in 6-well plates. Adjust the volume of reagents in proportion to the bottom area of the plate, as shown by examples in Table 2.*

**Table 2.** KSHV reactivation reagents by plate size

Plate type*	96-well plate	6-well plate	10-cm plate
Number of cells to seed per plate (well)	0.5 × 10^5^ cells/mL	5 × 10^5^ cells/mL	5 × 10^6^ cells/mL
Volume of Dox needed for 5 μg/mL per plate (well)	0.2 μL	3 μL	10 μL
Volume of NaBu needed for 1 mM per plate (well)	0.2 μL	3 μL	10 μL
Total volume of iSLK media	200 μL	3 mL	10 mL

*Use black plates (e.g., Greiner Bio-One, catalog number: 655090) and phenol-free iSLK cell media when seeding for automated plate reader experiments.

1. Prepare the tissue culture hood by cleaning surfaces with 70% ethanol.

2. Prepare reactivating reagent solutions (see Recipes 6 and 7).

3. Prewarm iSLK media and trypsin to 37 °C.

*Note: Do not seed cells using iSLK selection media.*

4. Retrieve cells from the 37 °C incubator. Check cell confluency and viability under the microscope.

**Critical:** At this point, cells must be properly selected with 50 μg/mL of G418, 1 mg/mL of hygromycin, and 1 μg/mL of puromycin (see section D) before being seeded for reactivation.

5. Aspirate the cell media using a 2-mL aspirating pipette.

6. Wash cells 1× with PBS (see Table 1).

7. Aspirate PBS from plates using a 2-mL aspirating pipette.

8. Trypsinize cells for 5 min in the 37 °C incubator. Be sure to coat the entire plate with trypsin (see Table 1).

9. Once cells have detached from the plate, resuspend cells in iSLK media (see Table 1).

a. Ensure cells are fully resuspended and no cell clumps are visible.

10. Transfer cell suspension into a 15 or 50 mL centrifuge tube.

11. In an Eppendorf tube, mix 10 μL of cell suspension with 10 μL of trypan blue. Add 10 μL of this mixture into a hemocytometer slide and count the number of live cells.

12. Plate iSLK cells at 5 × 10^5^ cells/mL for mock reactivation and KSHV reactivation. Swirl the cell suspension in the plates four times in a side-to-side motion and then four times in an up-and-down motion to evenly distribute cells.

**Critical:** Please allow cells ≥12 h to adhere to plates prior to KSHV reactivation.

13. The following day, reactivate cells with 5 μg/mL Dox and 1 mM NaBu.

a. For Mock reactivation, add PBS instead of reactivating reagents.

b. Dox and NaBu can be added directly into the media on the plate or added into fresh iSLK media and used to replace media in the wells from initial seeding.

c. Note the time of reactivation to reach the appropriate time point.

**Caution:** At this point, take BSL2 precautions as reactivated cells contain infectious KSHV. Disinfect pipettes, pipette tips, and tissue culture hood area with 20% bleach (1.57% sodium hypochlorite) for 10 min (see Recipe 10).

**Figure 1.**
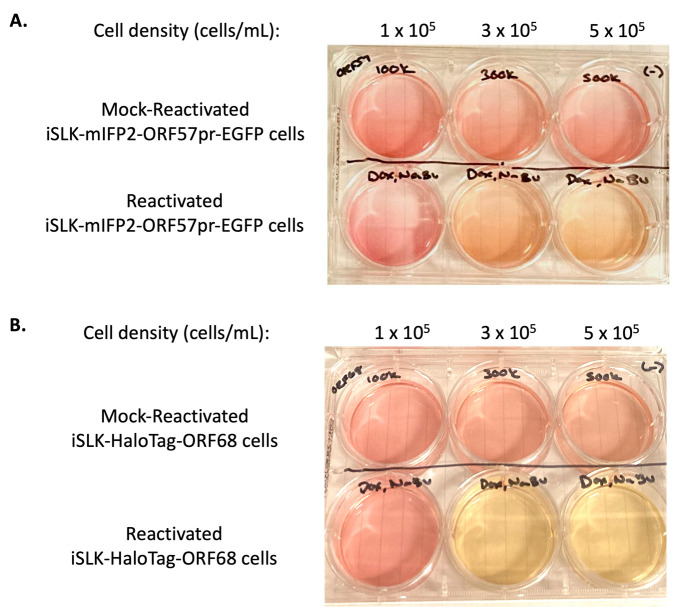
Comparison of iSLK cell media color based on cell density and reactivation. iSLK-BAC16-mIFP2-ORF57pr-EGFP cells (A) and iSLK-BAC16-HaloTag-ORF68 cells (B) were seeded at 1 × 10^5^, 3 × 10^5^, and 5 × 10^5^ cells/mL. iSLK cells were then mock reactivated with PBS (top row) or reactivated with 5 μg/mL Dox and 1 mM NaBu (bottom row) for 72 h. Mock-reactivated cell media is pink/orange, whereas reactivated cell media changes into a light orange/yellow color.

**Figure 2.**
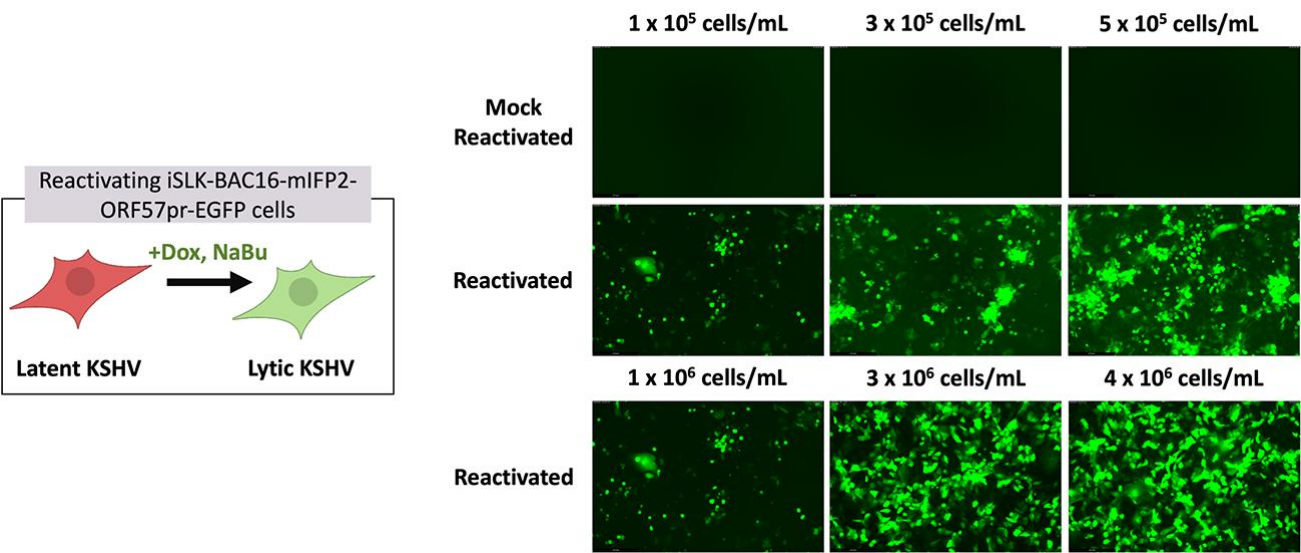
Comparison of iSLK reporter expression and expected cell death based on cell density and reactivation. Representative images of GFP expression in iSLK-BAC16-mIFP2-ORF57pr-EGFP cells seeded at 1 × 10^5^, 3 × 10^5^, and 5 × 10^5^ in 6-well plates and 1 × 10^6^, 3 × 10^6^, and 4 × 10^6 ^cells/mL in 10-cm plates. iSLK cells were mock reactivated with PBS (top row) or reactivated with 5 μg/mL Dox and 1 mM NaBu (bottom two rows) for 72 h. Increased numbers of rounded, detached cells and GFP expression are observed in reactivated iSLK-BAC16-mIFP2-ORF57pr-EGFP cells in a cell density–dependent manner. GFP expression in each individual well was detected using the LAS X software. Experiments were performed using two independent biological replicates.

**Figure 3.**
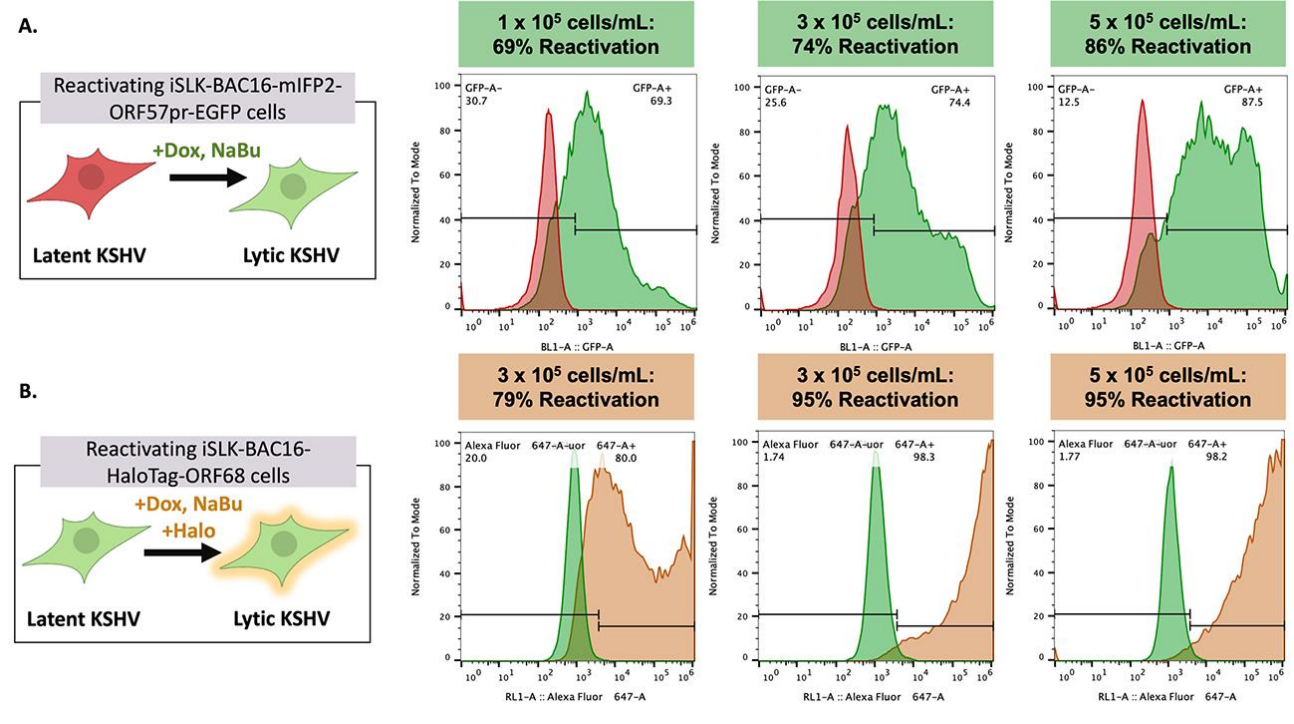
Kaposi’s sarcoma-associated herpesvirus (KSHV) reactivation rates vary depending on cell density and cell line. Representative flow plots of GFP expression in iSLK-BAC16-mIFP2-ORF57pr-EGFP cells (A) and Alexa Fluor 646 expression in iSLK-BAC16-HaloTag-ORF68 cells (B) seeded at 1 × 10^5^, 3 × 10^5^, and 5 × 10^5^ cells/mL in 6-well plates. iSLK cells were mock reactivated with PBS or reactivated with 5 μg/mL Dox and 1 mM NaBu for 72 h. For iSLK-BAC16-HaloTag-ORF68 cells, 20 nM of Janelia Fluor 646 Halo ligand was added to both mock and reactivated cells for ~1 h at 37 °C and 5% CO_2_. Reactivation rates increase in a cell density–dependent manner. Flow data were analyzed using FlowJo software with mock-reactivated samples as controls. Experiments were performed using two independent biological replicates, with ~10% variation in reported reactivation rates between replicates.

14. Culture KSHV reactivated iSLK cells until the desired time point is reached. For time points ≥48 h post-reactivation, there is an observable and distinct change in color from mock-reactivated iSLK cell media and reactivated iSLK cell media (Figure 1), corresponding to cell density and extent of KSHV reactivation (Figures 2 and 3).

**F. Flow cytometry for KSHV reactivated iSLK cells**

*Notes:*

*1. All steps are performed in a laminar flow hood unless otherwise noted.*

*2. Flow cytometry is used to quantify KSHV reactivation rates in mock and reactivated iSLK cells (Figure 3). Special training may be required to operate the flow cytometer. The following protocol is for flow cytometry of iSLK cells (with fluorescent reporters for reactivation) in 6-well plates. Adjust the volume of reagents in proportion to the bottom area of the plate, as shown by examples in Table 1. For iSLK cells without fluorescent reporters for reactivation, cells can be permeabilized with 100% methanol and immuno-stained with antibodies against lytic KSHV genes (e.g.*, KSHV ORF45, k8.1, *etc.)*

**Caution:** Use BSL2 precautions as reactivated iSLK cells contain infectious KSHV. Disinfect everything that comes into contact with KSHV (media, wash buffer, pipette tips, pipettes, and tissue culture plates) with 20% bleach (1.57% sodium hypochlorite) for 10 min (see Recipe 10).

1. For iSLK cells containing HaloTags, incubate both mock and reactivated cells with 20 nM Janelia Fluor 646 Halo Ligand for 30 min to overnight at 37 °C prior to flow or FACS experiments.

2. Once the desired time point is reached, prepare mock and reactivated cells for flow cytometry. Retrieve cells from the 37 °C incubator and check both cell viability and fluorescent reporter expression under the microscope.

*Note: By 48 h post-reactivation, ~1/5 or more of reactivated cells will round off and die, with increased cell death observed during later time points (≥72 h).*

3. Discard the cell media in 20% bleach for 10 min (see Recipe 10).

4. Wash cells 1× with 1 mL of PBS.

5. Discard PBS from plates into 20% bleach for 10 min.

6. Trypsinize cells with 1 mL of trypsin for 5 min in the 37 °C incubator, making sure to coat the entire plate with trypsin.

7. Once cells have detached from the plate, resuspend cells in 1 mL of iSLK media. Ensure cells are fully resuspended and no cell clumps are visible.

8. Transfer cell suspensions into a 15 mL centrifuge tube. Centrifuge cells at 500× *g* for 5 min.

9. Discard supernatant in 20% bleach for 10 min.

10. Wash cells 2× with 1 mL of PBS by centrifuging cells at 500× *g* for 5 min, discarding supernatant in 20% bleach for 10 min.

11. Resuspend cells in 1 mL of 4% PFA for 15 min at room temperature.

12. Wash cells 2× with 1 mL of PBS by centrifuging cells at 500× *g* for 5 min, discarding supernatant in 20% bleach for 10 min.

13. Resuspend cell pellets in ~500 μL to 1 mL of PBS and transfer into flow tubes for flow cytometry.

14. Collect data on 20,000 events using the Attune flow cytometer software.

a. Set up dot-plots for side-scatter (SSC-A) and forward scatter (FSC-A).

b. Set up histograms for fluorescent reporters or fluorophore-conjugated antibodies.

i. For iSLK-BAC16-HaloTag-ORF68 cells: use blue laser 1 (BL1) for GFP and red laser one (RL1) for Alexa Fluor 646.

ii. For iSLK-BAC16-mIFP2-ORF57pr-EGFP cells: use BL1 for GFP and RL1 for mIFP2.

c. Use mock-reactivated iSLK cells as controls for sample gating and to adjust laser detector voltages.

i. Control samples should have a uniform histogram ≤10^3^.

15. Export .fsc files and analyze data using FlowJo (see Data analysis).

16. Disinfect flow samples in 20% bleach for 10 min.

**G. Plate reader assessment of KSHV reactivated iSLK cells**

*Notes:*

*1. All steps are performed in a laminar flow hood unless otherwise noted.*

**Figure 4.**
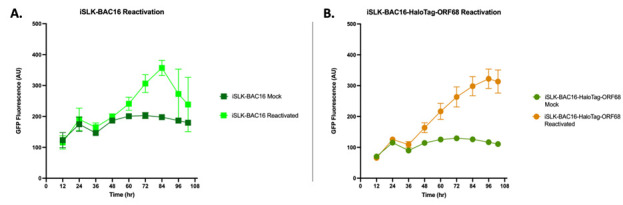
GFP fluorescence of Kaposi’s sarcoma-associated herpesvirus (KSHV) reactivated iSLK cells. iSLK-BAC16 (A) and iSLK-BAC16-HaloTag-ORF68 cells (B) were seeded at 0.5 × 10^5^ cells/mL in phenol-free iSLK media in black 96-well plates. iSLK cells were then mock reactivated with PBS or reactivated with 5 μg/ml Dox and 1 mM NaBu. Cells were placed in an automatic plate reader for ~108 h at 37 °C and 5% CO_2_. GFP expression of KSHV reactivated cells increases over time, with a significant difference observed as early as ~36–48 h post-reactivation. Each experiment represents a single biological replicate with three technical replicates. Bars represent mean values, and the error bars represent SEM.

*2. The following protocol is for the use of a temperature-controlled, automated plate reader (connected to CO_2_) to assess KSHV reactivation of iSLK cells with fluorescent reporters for reactivation (Figure 4). To minimize auto-fluorescence, we recommend using phenol-free iSLK media (see Recipe 9) and the use of black, tissue culture–treated 96-well plates. To scale up, adjust the volume of reagents in proportion to the bottom area of the plate, as shown by examples in Table 2, being sure to use black, tissue culture–treated plates.*

**Caution:** Use BSL2 precautions as reactivated iSLK cells contain infectious KSHV. Disinfect everything that comes into contact with KSHV (media, wash buffer, pipette tips, pipettes, and tissue culture plates) with 20% bleach for 10 min (see Recipe 10).

1. Prepare the tissue culture hood by cleaning surfaces with 70% ethanol.

2. Prepare reactivating reagent solutions (see Recipes 6 and 7) and phenol-free iSLK cell media (see Recipe 9).

3. Prewarm trypsin to 37 °C.

*Note: Do not seed cells using iSLK selection media.*

**Critical:** Use of phenol-free iSLK cell media drastically reduces artificially high fluorescence readings due to auto-fluorescent behavior of iSLK cell media (containing phenol red).

4. Retrieve cells from the 37 °C incubator. Check cell confluency and viability under the microscope.

**Critical:** At this point, cells must be properly antibiotically selected with 50 μg/mL of G418, 1 mg/mL of hygromycin, and 1 μg/mL of puromycin (see section D) before seeding for reactivation.

5. Aspirate the cell media using a 2-mL aspirating pipette.

6. Wash cells 1× with PBS (see Table 1).

7. Aspirate PBS from plates using a 2-mL aspirating pipette.

8. Trypsinize cells for 5 min in the 37 °C incubator. Be sure to coat the entire plate with trypsin (see Table 1).

9. Once cells have detached from the plate, resuspend cells in iSLK media (see Table 1). Ensure cells are fully resuspended and no cell clumps are visible.

10. Transfer cell suspension into a 15 or 50 mL centrifuge tube.

11. In an Eppendorf tube, mix 10 μL of cell suspension with 10 μL of trypan blue. Add 10 μL of this mixture into a hemocytometer slide and count the number of live cells.

12. Centrifuge cells at 500× *g* for 5 min.

13. Aspirate the cell media using a 2-mL aspirating pipette and wash the cell pellet 2× with PBS by centrifugation.

14. Resuspend the cell pellet in phenol-free iSLK cell media.

15. Plate cells at 0.5 × 10^5^ cells/mL in black, tissue culture–treated 96-well plates for mock reactivation and KSHV reactivation. Swirl the cell suspension in the plates four times in a side-to-side motion and then four times in an up-and-down motion to evenly distribute cells.

**Critical:** Avoid seeding cells in the outer-most wells (A1–12, A1–H1, A12–H12, and H1–12) as readings can be inconsistent. Wells should contain 200 μL of phenol-free iSLK media, as time courses can result in ~50 μL evaporation per well. All wells should contain media, even if data will not be recorded in said wells, to minimize evaporation of media in wells containing samples.

**Critical:** Please allow cells ≥12 h to adhere to plates prior to KSHV reactivation.

16. The following day, reactivate cells with 5 μg/mL of Dox and 1 mM NaBu.

a. For mock reactivation, add PBS instead of reactivating reagents.

b. Note the time of reactivation to reach the appropriate time point.

**Caution:** At this point, take BSL2 precautions as reactivated cells contain infectious KSHV. Disinfect pipettes, pipette tips, and tissue culture hood area with 20% bleach for 10 min (see Recipe 10).

17. Place the 96-well plate in a temperature-controlled, automated plate reader with 5% CO_2_ to collect fluorescent readings on KSHV reactivated iSLK cells until the desired time point is reached (Figure 4).

a. Collect data based on fluorescent reporters of iSLK cells. For GFP: Use 488 nm for the excitation wavelength and 510 nm for the emission wavelength.

**Critical:** Take readings from the top of the well. Readings from the bottom of the well without calibrating measurements to the specific plate being used lead to inaccurate fluorescence readings.

## Data analysis

**Flow cytometry quantification of KSHV reactivation in iSLK cells**

*Note: Flow cytometry data collected on mock reactivated and KSHV reactivated iSLK-BAC16 cells can be analyzed using the FlowJo software (this will require a license). Flow cytometry data of technical replicates (triplicates) can be used to quantify variability within a biological sample. Analysis of data across experiments can be used to quantify the variability between biological replicates. We include mock-reactivated controls for all experiments and ensure laser settings are consistent across flow cytometry runs.*

**Figure 5.**
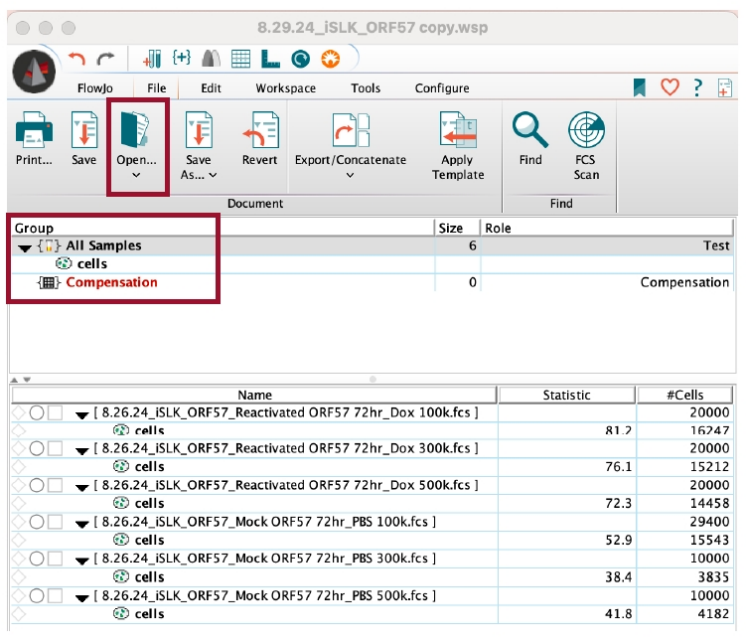
FlowJo interface for flow cytometry analysis. The FlowJo software allows users to analyze flow cytometry data. In the workspace, append flow data (.fsc files) by either dragging files into the workspace or by opening the files through the FlowJo interface. To apply gates or analyses from controls to the entire group, drag the analysis to *Group > All Samples.*

**Figure 6.**
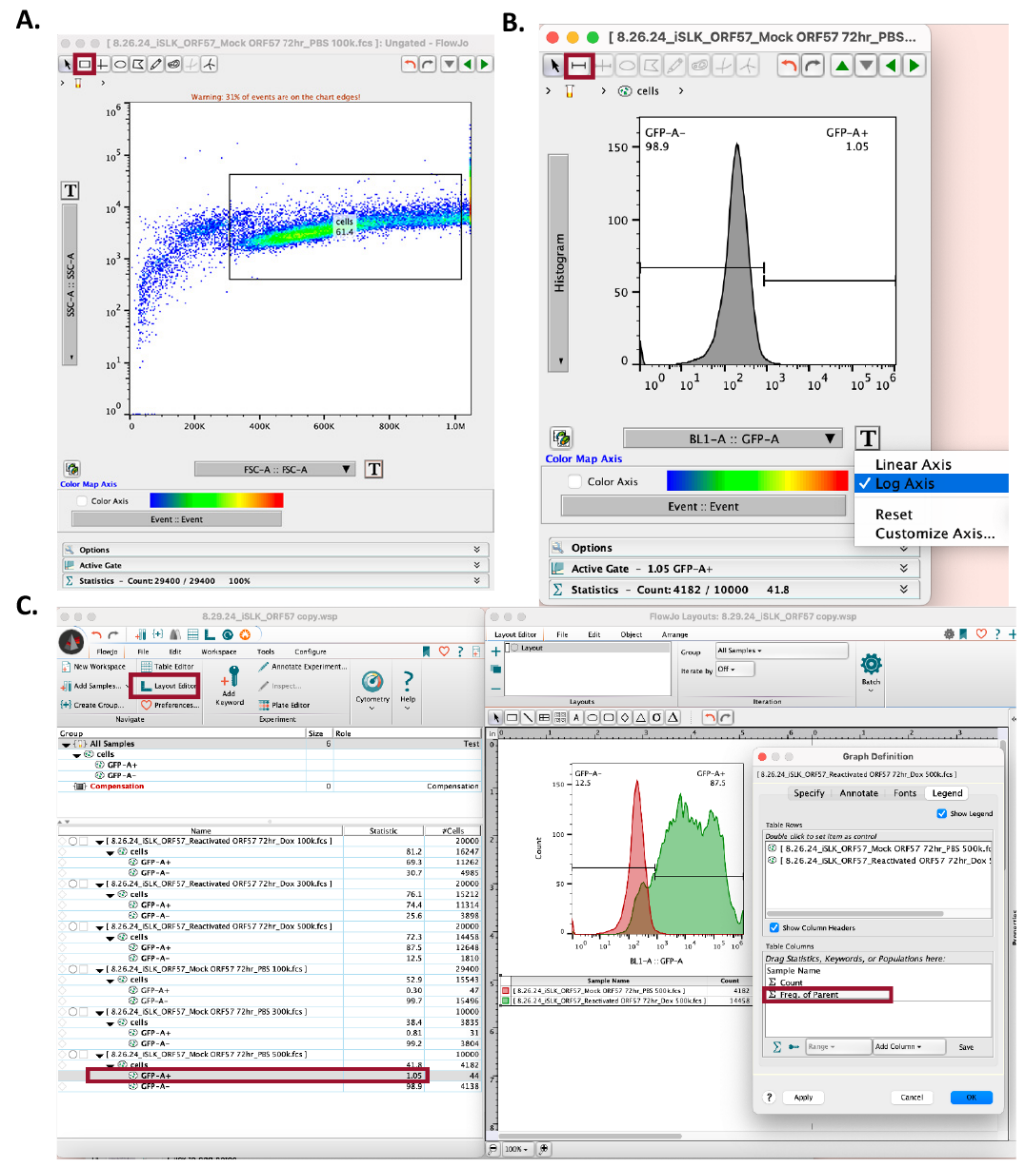
Using FlowJo to set gates and quantify Kaposi’s sarcoma-associated herpesvirus (KSHV) reactivation. (A) In the workspace, open the mock-reactivated flow data. In the SSC-A vs. FSC-A dot plot, set gates using the square tool, avoiding cell debris (far-left) and cell clumps (far-right). (B) In the mock-reactivated data set, change the y-axis to *Histogram* and the x-axis to *log axis*, and the sample’s corresponding fluorescent reporter (e.g., *BL1-A* for GFP). Use the bisector tool to set the gate at the right side of the histogram. (C) Open *Layout Editor* to visualize and compare KSHV reactivation in mock-reactivated vs. reactivated samples. Drag the cell analyses for each sample to the layout editor and overlay them. Click on the figure legend to add the reactivation rate statistics by dragging the sample analysis to the *Table Columns* space and selecting OK. Data used in this figure is representative of KSHV reactivation in iSLK-BAC16-mIFP2-ORF57pr-EGFP cells.

1. Open the FlowJo software.

2. Open the data files by going to *File > Open* and selecting the corresponding .fsc files. The workspace will now show all the data files to be analyzed (Figure 5).

3. Open the mock reactivated .fsc file.

a. Set the y-axis to *SSC-A::SSC-A* and the x-axis to *FSC-A::FSC-A* (Figure 6A).

b. Using the square tool, set gates to include the iSLK cell population (Figure 6A).

i. Exclude cells with very low SSC-A and FSC-A values, as this represents cell debris.

ii. Exclude cells with very high SSC-A and FSC-A values, as this represents cell doublets or cell clumps.

4. Drag the mock reactivated dot plot analysis to the corresponding reactivated .fsc file to copy and apply this analysis to the group (Figure 5).

a. Ensure the gate set by the mock reactivated cell sample includes the iSLK cell population in the reactivated iSLK cell samples.

5. In both the mock reactivated and reactivated .fsc files, change the y-axis into a *histogram* and the x-axis to the appropriate laser [this will be based on the iSLK cell fluorescent reporter(s) or fluorophore-conjugated antibodies]. Set the x-axis to *log axis* (Figure 6B).

a. For mock reactivated cell samples, there should be a uniform peak around 10^3^.

6. In the mock reactivated .fsc file, use the bisector tool (⊢ ⊣) to set the gate to the right of the peak (Figure 6B).

7. Drag the mock reactivated histogram analysis to the corresponding reactivated .fsc files to copy and apply this analysis to the group (Figure 5).

8. Open the layout editor by going to *FlowJo > Layout Editor* (Figure 6C).

9. Drag the mock reactivated histogram analysis to the layout editor.

10. Merge the analysis by placing the reactivated histogram analysis on top of the mock reactivated analysis in the layout editor (Figure 6C).

11. Locate the flow cytometry plot legends, then click on the colored boxes to adjust sample colors based on the sample’s fluorescent reporter or fluorophore-conjugated antibody.

12. Open the flow cytometry plot legend to set the frequency of the parent (Figure 6C). Drag the positive reporter statistical analysis (e.g., *GFP+)* to the graph legend table columns. Click OK.

13. Export the flow cytometry plot by going to *File > Export Image > PNG.*

14. Save the workspace as a .wsp file by going to *File > Save As > Save as Workspace (WSP).*

## Validation of protocol

Parts of this protocol have been used and validated in the following research article(s):

• Brulois et al. [9] Construction and Manipulation of a New Kaposi’s Sarcoma-Associated Herpesvirus Bacterial Artificial Chromosome Clone. *J. Virol*. (Figure 3, Figure 4D, and Figure 5B).

• Morgens et al. [10] A Two-tiered functional screen identifies herpesviral transcriptional modifiers and their essential domains. *PLOS Pathog.* (Supplemental Figure 2D, E and Figure 3C).

• McCollum et al. [11] The viral packaging motor potentiates Kaposi’s sarcoma-associated herpesvirus gene expression late in infection. *PLOS Pathog.* (Supplemental Figure 2A and Figure 2D).

**Figure 7.**
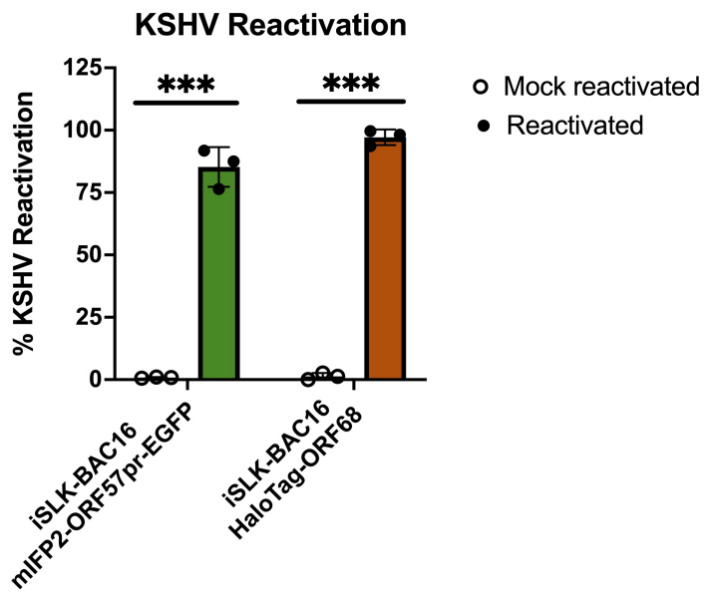
Flow cytometry quantification of Kaposi’s sarcoma-associated herpesvirus (KSHV) reactivation. iSLK-BAC16-mIFP2-ORF57pr-EGFP cells and iSLK-BAC16-HaloTag-ORF68 cells were seeded at 5 × 10^5^ cells/mL in 6-well plates. iSLK cells were then mock reactivated with PBS or reactivated with 5 μg/ml Dox and 1 mM NaBu for 72 h. For iSLK-BAC16-HaloTag-ORF68 cells, 20 nM of Janelia Fluor 646 Halo ligand was added to both mock and reactivated cells for ~1 h at 37 °C and 5% CO_2_. Flow data were analyzed using FlowJo software with mock-reactivated samples as controls. KSHV reactivation rates were determined based on positive fluorescence expression of the lytic marker compared to controls and analyzed using multiple, unpaired t-tests using GraphPad prism. *** denotes p-values < 0.0005. For each experiment, a circle represents a biological replicate conducted with three technical replicates. Bars represent mean values, and the error bars represent SEM.

KSHV reactivation data for iSLK-BAC16-mIFP2-ORF57pr-EGFP and iSLK-BAC16-HaloTag-ORF68 cells were collected using flow cytometry and quantified using the FlowJo software, with mock-reactivated samples serving as controls. For iSLK-BAC16-mIFP2-ORF57pr-EGFP cells, GFP expression was used as a read-out for KSHV reactivation. For iSLK-BAC16-HaloTag-ORF68 cells, Alexa-646 expression (orange/far-red) was used as a read-out for KSHV reactivation. KSHV reactivation rates across biological replicates were then plotted on GraphPad Prism and analyzed using multiple, unpaired t-tests (Figure 7). Using this protocol, KSHV reactivation is consistent and robust.

## General notes and troubleshooting

**General notes**

In this protocol, we use iSLK-BAC16-HaloTag-ORF68 and iSLK-BAC16-mIFP2-ORF57pr-EGFP cells as example cell lines for reactivating KSHV, which are comparable for detecting latent to lytic phases of KSHV reactivation. The procedures outlined in this protocol also apply to other iSLK-BAC16 cells and their derivatives.

1. iSLK-BAC16 cells are adherent, allowing determination of cell confluency by eye under a microscope (e.g., 80% confluency equates to 80% of the plate covered by cells).

2. iSLK-BAC16 cells divide every 24 h and prefer being confluent (cells grow slowly when sparse). Generally, iSLK-BAC16 cells at ≥80% confluency (1.4–1.5 × 10^4^ cells/cm^2^) can be split 1:4 or 1:5 every 2–3 days.

3. When iSLK-BAC16 cells become overcrowded, KSHV reactivation rates can decrease. Do not allow iSLK-BAC16 cell cultures to exceed 100% confluency.

a. In a 15-cm plate, do not exceed a cell density of 2 × 10^7^ cells/mL.

4. To minimize genetic variation and prevent drift, do not culture iSLK-BAC16 cells beyond one month from thawing (maximum of 12–14 passages).

5. This protocol is adaptable for KSHV reactivation at any scale. From one well of a 6-well plate, seeded at 5 × 10^5^ cells/mL, ~80%–90% of KSHV reactivation can be expected.

6. The following are recommended cell densities for 6-well and 10-cm plates:

a. For 6-well plates: seed 5 × 10^5^ cells/mL.

b. For 10-cm plates: 5 × 10^6^ cells/mL.

**Troubleshooting**

**Problem 1:** Low cell viability during antibiotic selection.

**Possible cause:** Improper antibiotic selection of cells results in loss of the Dox-inducible RTA system.

**Solution 1:** Use fresh cell stocks or early-passage cells, as they may contain higher numbers of cells harboring the Dox-inducible RTA system.

**Solution 2:** Cell density maintained at ≥80% confluency is recommended during initial antibiotic selection to maintain sufficient numbers of viable cells.

**Solution 3:** Selecting cells with 1 mg/mL of hygromycin may quickly kill cells lacking the Dox-inducible RTA system. Adjust the concentration of hygromycin, starting with 200 μg/mL and incrementally increasing hygromycin concentrations until a final concentration of 1 mg/mL is reached.

## Supplementary information

The following supporting information can be downloaded here:

1. Figure S1. Effects of Dox on KSHV reactivation rates
